# Changes of Symptoms of Anxiety, Depression, and Fatigue in Cancer Patients 3 Months after a Video-Based Intervention

**DOI:** 10.3390/ijerph20206933

**Published:** 2023-10-17

**Authors:** Sina Schlecht, Sven Neubert, Karin Meng, Antonia Rabe, Elisabeth Jentschke

**Affiliations:** Comprehensive Cancer Center Mainfranken, University Hospital Würzburg, 97080 Würzburg, Germany; sina.schlecht@stud-mail.uni-wuerzburg.de (S.S.); sven.neubert@stud-mail.uni-wuerzburg.de (S.N.); meng_k@ukw.de (K.M.); rabe_a1@ukw.de (A.R.)

**Keywords:** cancer, psycho-oncology, eHealth, supportive care intervention, psychoeducation, mind–body intervention, distress

## Abstract

During the COVID-19 pandemic, social distancing restricted psycho-oncological care. Therefore, this secondary analysis examines the changes in anxiety, fear of progression, fatigue, and depression in cancer patients after a video-based eHealth intervention. We used a prospective observational design with 155 cancer patients with mixed tumor entities. Data were assessed before and after the intervention and at a three-month follow-up using self-reported questionnaires (GAD-7, FOP-Q-SF, PHQ-8, and EORTC QLQ-FA12). The eight videos included psychoeducation, Acceptance and Commitment Therapy elements, and yoga and qigong exercises. The results showed that three months after finishing the video-based intervention, participants showed significantly reduced fear of progression (d = −0.23), depression (d = −0.27), and fatigue (d = −0.24) compared to the baseline. However, there was no change in anxiety (d = −0.09). Findings indicated marginal improvements in mental distress when using video-based intervention for cancer patients for up to three months, but long-term effectiveness must be confirmed using a controlled design.

## 1. Introduction

Every second patient with cancer has to deal with the psychological burden of their cancer diagnosis and from the consequences of cancer treatment [1]. One in four cancer patients show depressive symptoms [2]. Moreover, half of the patients with cancer suffer from cancer-related fatigue [1,3]. Up to 40% of anxiety symptoms are described among long-term cancer survivors [4]. Therefore, almost one-third of cancer patients use psycho-oncological support [5] to deal with cancer and its psychological consequences.

Additionally, the COVID-19 pandemic increased the psychological burden of cancer patients [6,7]. A meta-analysis [6] showed that the prevalences of depression and anxiety in cancer patients during the pandemic were 37% and 38%, respectively. Furthermore, there was a considerable level of comorbidity, with a prevalence of 10%. Compared to healthy controls, cancer patients had higher anxiety levels. However, substantial heterogeneity emerged that might be due to the assessment method, as included studies used different screening questionnaires. Furthermore, a recent study on women with breast cancer showed an increased risk of developing affective disorders, like anxiety disorders and depressive symptoms, due to the pandemic [7]. One reason could be that cancer patients have been defined as a vulnerable group in the COVID-19 pandemic [7] due to the immune-suppressive effects of cancer treatment [8]. Cancer patients are even threatened indirectly due to interruptions to running tumor therapies or delayed cancer diagnosis [7] as a result of lockdown measures and shutting down standard operations in hospitals [7,9,10]. Due to this burden, there is an imminent need for psycho-oncological care.

Since the beginning of the pandemic, psycho-oncological care has met several challenges. First, the necessity of social distancing has led to a reduction in usual psycho-oncological care. In order to meet the high demand for psychological support combined with contact limitations, digital psychological support became more important than ever.

Digital interventions are part of eHealth interventions [11], in which digital psycho-oncological interventions have been determined to have the potential to help reduce the gap in mental health services [12]. Before the COVID-19 pandemic, digital interventions were subject to several psycho-oncological trials. A meta-analysis found significant reductions in depression and fatigue due to internet-based psychoeducation for patients with cancer [13]. In addition, several studies reported promising effects of digital psychological interventions on anxiety and depression [14,15,16]. However, studies often included only a few tumor entities, and male participants were underrepresented [16,17,18,19,20]. Moreover, many researchers have only focused on the short-term effects of digital psycho-oncological interventions [13,16], and some reported that long-term examinations exhibited no effects [14,21]. Therefore, there needs to be a greater understanding of how long the benefits of digital psycho-oncological interventions last.

Regarding the wide range of eHealth applications, studies have shown that web-based applications are suitable for psycho-oncological interventions [22,23,24]. A recent review shows that video-based intervention studies often target cancer screening and treatment and that only a few interventions address coping [25]. A survey concluded that anxiety symptoms and coping abilities are highly relevant topics for psycho-oncological eHealth applications [22].

Overall, there is a need to examine video-based interventions targeting coping strategies, integrating more tumor entities and follow-up observations. Therefore, our team established a video-based intervention focusing on psychoeducation and physical exercises to improve coping with symptoms of anxiety, depression, and fatigue in a previous study [26]. Psychoeducation has been proven to have positive effects on anxiety [27], depression [27], and fatigue [28]. Furthermore, we used methods with proven effectiveness [29,30,31,32,33,34], like Acceptance and Commitment Therapy (ACT), as well as mindfulness-based therapy (MBT). Moreover, physical exercises like yoga and qigong were selected to complete our intervention [35,36,37,38]. However, this randomized controlled trial (RCT) showed no significant effects in terms of reported symptoms compared to a waiting control group at the end of treatment [26]. To confirm these results, the present observational study was carried out with a larger sample, evaluating the participants over three months (the waiting list group in the previous study underwent intervention and were included in the sample).

First, we hypothesized that the scores for anxiety, fear of progression, depression, and fatigue would improve significantly between the baseline and three months after the intervention.

Furthermore, we hypothesized no significant changes in anxiety symptoms, fear of progression, depression, and fatigue symptoms between the end of the intervention and the follow-up period.

Secondly, we investigated if participants who maintained video usage and exercised during the follow-up period had better anxiety, fear of progression, depression, and fatigue outcomes over time than those who stopped video usage and practice.

## 2. Materials and Methods

### 2.1. Setting and Sample

The prospective observational study was conducted at the University Hospital of Wuerzburg, Germany, Comprehensive Cancer Center Mainfranken (CCCMF). The Ethics Committee of the University of Würzburg approved the study (ref. 123/20-me). Reporting complies with common guidelines, e.g., [39].

Patients with malignant tumor disease, 18-years or older, and provided informed consent were included in the trial (inclusion criteria). In addition, participants were required to have internet access or at least a DVD-compatible device to watch the videos. There was no preselection concerning their current psychosocial distress. Exclusion criteria were severe physical or mental impairment and inadequate German language abilities.

Patients with diverse tumor entities were recruited from institutions within the CCCMF, like the interdisciplinary oncological therapy outpatient clinic (IOT), ambulance of psycho-oncology, and various oncological stations of the University Hospital of Wuerzburg. Written and verbal information about the study was provided before patients gave written consent. The video intervention comprised eight videos and lasted for four weeks. During this period, the waiting control group participants received no videos. The video intervention was offered to the control group afterwards. The participants completed questionnaires at baseline (T1), after the intervention group finished the video intervention, and when the control group ended their video intervention (T2). The last survey was conducted after a follow-up period three months from the end of the video intervention of each group (T3). Since both groups attended the video intervention, we combined both groups for this secondary observational study to examine the changes over time.

The sample size calculation of the previous study [26] determined the sample size.

### 2.2. Intervention

Participants had access to the CCCMF website, on which two videos were provided weekly. Participants received weekly information via email when new videos were available. The length of the videos was about twelve to thirty minutes. Patients could watch the videos repeatedly and save them via download. Therefore, the video usage was accessible at the will of participants. In addition, the participants could watch the videos they were interested in at their own pace. Therefore, the flooding of information should have been avoided. Furthermore, self-determined video selection might have reduced dropouts [12]. During the follow-up period, participants obtained three reminder emails to remind them about the availability of the videos.

A psycho-oncologist performed the video intervention. In line with psychoeducation, each video included knowledge about a central topic and appropriate physical exercise. Therefore, information on signs and symptoms was given, as well as guidance and psycho-oncological tools to improve coping. Besides psychoeducation, some aspects inspired by MBT and ACT were used. Most integrated physical exercises were based on Hatha yoga, which has its roots in mindfulness-based stress reduction from Kabat-Zinn [40]. Moreover, one physical exercise was based on qigong, and one relaxation exercise was provided. A more detailed summary of the video sequences is reported elsewhere [26].

### 2.3. Measure

- Anxiety

In order to assess anxiety symptoms, we used the Generalized Anxiety Disorder scale (GAD-7), which is a questionnaire with good validity and reliability [41]. GAD-7 consists of seven items that refer to anxiety symptoms within the last two weeks. The items address core symptoms of anxiety disorders (e.g., nervousness, uncontrollable worries, etc.) according to DSM-IV criteria. Items can be answered on a 4-point Likert scale (0 = not at all; 1 = on individual days; 2 = more than half the days; 3 = nearly every day). Items are summed up (general anxiety score range: 0–21), with higher scores indicating a higher expression of anxiety symptoms. Cut-off points of 5, 10, and 15 can be interpreted as representing mild, moderate, and severe levels of anxiety on the scale, respectively. The seven items had a good internal consistency (α = 0.88).

- Fear of progression

To assess fear of progression, we used the short form of the fear of progression questionnaire (FoP-Q-SF), which is a valid and reliable instrument for cancer patients [42,43]. FoP-Q-SF comprises 12 items rated on a 5-point Likert scale (1 = never; 2 = seldom; 3 = sometimes; 4 = often; 5 = very often). Items are combined into a sum score (range: 0–60), with higher scores indicating a higher fear of progression. A score of 34 or higher indicates a dysfunctional level of fear of progression. Cronbach α for the scale was 0.87.

- Depression

The Patient Health Questionnaire-8 (PHQ-8) was used to measure depressive symptoms [44]. Patients rate eight items that refer to symptoms of a depressive episode, according to DSM-IV criteria (e.g., anhedonia, depressed mood, etc.), that have occurred during the last two weeks on a 4-point Likert scale (0 = not at all; 1 = on individual days; 2 = more than half the days; 3 = nearly every day). Items are summed to a score from 0 to 24, with higher scores indicating more severe depressive symptoms. Scores of 5, 10, 15, and 20 represent mild, moderate, moderately severe, and severe depression, respectively [45]. Cronbach α for the scale was 0.83.

- Fatigue

Cancer-related fatigue was assessed with the fatigue scale of the European Organization For Research and Treatment of Cancer Quality of Life Questionnaire (EORTC QLQ-FA12). The questionnaire comprises twelve items of fatigue’s physical (e.g., lacked energy, feel sleepy during the day, etc.), cognitive (e.g., feel confused, have trouble thinking clearly, etc.), and emotional aspects (e.g., feel helpless, frustrated, etc.), rated on a 4-point Likert scale. The total score is transformed to a scale ranging from 0 to 100, with higher scores representing more severe fatigue symptoms [46,47]. The scale showed a high internal consistency (α = 0.92).

- Treatment adherence

Moreover, patients were asked about video usage and exercise practice during the follow-up period (Table 1). Video use and practice frequency were assessed with two items. The reasons for maintaining or quitting video usage were also asked about, with multiple response options.

### 2.4. Statistical Analyses

Analyses were performed using IBM SPSS Statistics Version 27 (IBM Corporation, New York, USA). To analyze the hypotheses on changes between baseline and follow-up and post-intervention and follow-up, we used paired *t*-tests. In addition, standardized effect size (SES; small = 0.2, medium = 0.5, large = 0.8) and accompanying 95% confidence intervals (CI) were determined. Questionnaires with missing values were excluded by pairwise deletion. For all statistical analyses, *p* < 0.05 was considered significant. No adjustment for multiple tests was planned. Therefore, we reported the exact *p*-values.

To explore the relationship between our intervention outcomes and exercise practice or video usage during the follow-up, we calculated an analysis of variance with repeated measures (ANOVA). Data were adjusted for statistical outliers. We explored outliers by calculating box-and-whisker plots for each group and defined outliers as values greater or less than 1.5 × the interquartile range. The number of excluded outliers varied between 0 and 4 for the analyses.

## 3. Results

### 3.1. Sample

**Figure 1:**
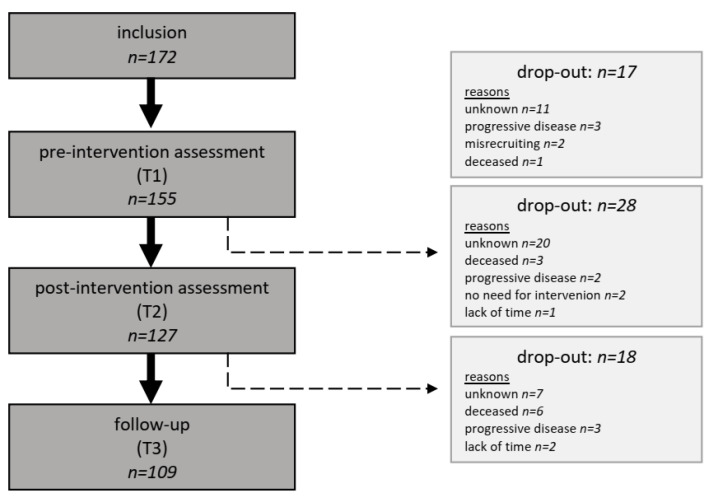
Participant flow.

From June 2020 to September 2020, a total of 172 patients were recruited. Due to drop-outs (*n* = 17) before the first assessment, our initial sample comprised 155 patients. A further 46 patients were lost until follow-up. Therefore, our analysis refers to patients with data on all assessment points (*n* = 109). For details on the study flow, see Figure 1. Drop-out analysis was performed for the sociodemographic and clinical variables. The results indicate no systematic sample bias because of patient drop-out. However, significantly more dropouts had metastasis (*p* < 0.001), were under tumor therapy (*p* = 0.015), or received palliative care (*p* < 0.001) than the completers.

Table 2 provides information about the sociodemographic and clinical sample characteristics at the baseline. The mean age of the sample was 56.1 years (SD = 12.6). About two-thirds were female (68%). Seventy-five percent were married or living in a partnership. Seventy-eight percent had secondary or higher education. The most common tumor diagnoses were haemato-oncological tumors (36%) and breast cancer (30%), followed by gynecological tumor entities (8%) and colon cancer (8%). About a fifth of the participants had metastasis, and 16% had a cancer recurrence. In addition, 54% were under cancer-specific treatment, and 20% received palliative care. About 30% of participants had received cancer diagnoses during the last twelve months.

### 3.2. Changes in Anxiety, Depression and Datigue at 3-Month Follow-Up

Table 3 shows the results of changes between the baseline (T1) and follow-up (T3). There was a slight but non-significant reduction in anxiety symptoms (SES = −0.09, 95%-CI = −0.25 to 0.07). However, fear of progression was significantly reduced with a small effect size (SES = −0.23, 95%-CI = −0.36 to −0.10). Furthermore, significant small changes were found for symptoms of depression (SES = −0.27, 95%-CI = −0.44 to −0.11) and general fatigue (SES = -0.24, 95%-CI = −0.41 to −0.07). These results suggest a small improvement in symptoms of fear of progression, depression, and fatigue from video intervention, while anxiety symptoms remained unchanged.

Table 4 summarizes the results of short-term changes (T2 to T3). Compared with post-intervention values (T2), no significant changes were found for symptoms of anxiety (SES = −0.04, 95%-CI −0.16 to 0.08), depression (SES = −0.09, 95%-CI = −0.22 to 0.04), and fatigue (SES = −0.04, 95%-CI = −0.17 to 0.09) at the follow-up analysis (T3), which indicate stable values during the follow-up period. However, values for fear of progression declined significantly with a marginal effect size during the follow-up period (SES = −0.13, 95%-CI = −0.25 to −0.02).

### 3.3. Relation between Video Usage, Exercise Practice and Outcomes

Of the 109 participants, 67% (*n* = 73) continued using video sequences during the follow-up period. Of all participants, 9% (*n* = 10) used seven or eight videos, about 24% (*n* = 26) used four to six videos, and about 34% (*n* = 37) used one to three videos. One-third (*n* = 36) had not seen any of the videos during the follow-up period. Concerning the frequency and regularity, of those who continued video usage (*n* = 73), 4% (*n* = 3) used the videos several times per week, 26% (*n* = 19) used the videos once a week, and 67% (*n* = 49) used the videos rarely. The most popular reasons (*n* = 70) to continue video use were an improvement in coping with the disease (45.7%, *n* = 32) as well as recovering from sedation (45.7%, *n* = 32). On the other hand, the main reasons for quitting video usage (*n* = 36) were a lack of need due to general well-being (52.7%, *n* = 19) and a lack of time (33.3%, *n* = 12).

Ninety-three participants answered the questions about the integrated exercises. Thereof, about 6% (*n* = 6) conducted daily exercise, 23% (*n* = 21) performed the exercises several times a week, and 18% (*n* = 17) practiced once a week, while 38% (*n* = 35) performed the exercises more rarely. Therefore, 15% (*n* = 14) conducted none of the exercises during the follow-up period.

In order to examine the relationship between outcomes and video usage or exercise practice, participants who answered yes to continuing the usage of all or several video sequences were determined as “video-users” (*n* = 73). Those who failed to use any video sequence during the follow-up period were defined as “non-users” (*n* = 36). Moreover, participants who executed the exercises daily, several times per week, or once a week were summed up as “practicers” (*n* = 44). Finally, those who stopped exercise or performed them rarely were termed “non-practicers” (*n* = 49). Results of the exploratory group comparisons over time (ANOVA) are summarized in Supplementary Table S1. The ANOVA results showed no significant time-by-group interaction effects for all outcomes. Users and practicers showed higher levels of symptom burden than non-users or non-practicers; however, most group comparisons failed to show significance due to the sample size.

## 4. Discussion

This prospective observational study examined the improvements in symptoms of anxiety, fear of progression, depression, and fatigue three months after a video-based intervention in oncological patients with diverse tumor entities. Compared to the values before the intervention, symptoms of fear of progression, depression, and fatigue declined with small effect sizes. However, we did not find a significant reduction in anxiety symptoms. Thus, our first hypotheses were only partially confirmed. Secondly, as hypothesized, there were no significant changes in symptoms of anxiety, depression, and fatigue during the follow-up period. However, we could not confirm our sub-hypothesis on fear of progression, though the significant changes were very small.

To our knowledge, no study has conducted a video-based psychoeducational intervention for cancer patients with a similar design and a three-month follow-up observation. Nevertheless, several studies evaluating other digital interventions achieved similar results. For example, an RCT examined a sixteen-week web-based self-management program for breast cancer patients, integrating some videos into the program. There were significant small-to-medium effects on the fear of recurrence and fatigue at the end of the intervention. At two- and six-month follow-ups, both groups showed significant improvements, and the fear of recurrence was only lower for patients in the digital intervention group [48]. An RCT evaluating an internet-based intervention used psychoeducation and internet-based cognitive behavioral therapy for patients with breast, colorectal, and prostate cancer. They showed a significant reduction in depressive symptoms after a follow-up period of ten months, but no effects on anxiety symptoms were seen compared to standard care [19]. Another RCT, using a web-based tailored intervention for cancer survivors, examined the effects on anxiety, depression, and fatigue. There were significant small reductions in depression and fatigue after six months [49], but after twelve months, the intervention group no longer differed from the control group [21].

The results might imply the potential sustained effects of digital interventions on depressive symptoms and fatigue. However, the effects on anxiety are limited. Moreover, it is still unclear how long the effects of digital interventions last. Our observational period of three months is relatively short. Therefore, it is unclear whether our results would remain stable over longer periods. Therefore, further studies are warranted to examine the long-term effectiveness of digital interventions on anxiety, depression, and fatigue.

Overall, digital interventions show a large variety in duration and use of therapeutic elements. Our video-based intervention comprised several therapeutic elements and lasted for four weeks. A meta-analysis found more significant reductions in psychological stress in cancer patients through psychoeducational interventions at high frequency and longer duration [27]. However, it is unclear if these assumptions are valid for video interventions comprising psychoeducation and (yoga) exercises. Concerning our mixed intervention, it is impossible to determine which elements of our video intervention contributed to the changes. Therefore, future video interventions might focus on one therapeutic element to gain better evidence. While there are indications that an interactive live-guided intervention might improve outcomes [50], our video intervention included no interactive tool, except contact via email, to link patients and providers. Therefore, there was a lack of personal contact, limiting questions that could be asked when they occurred. Patients often remarked on the lack of interaction when they gave their written feedback on our intervention. Following Leykin et al. [12], digital interventions cannot fully replace in-person psycho-oncological care. Therefore, future digital interventions might integrate a concept with more opportunities to link patients and therapists. As recommended in [12], we integrated skill-building and exercise practice into our intervention to improve self-management and coping. Nevertheless, we found no significant relation between video usage or practice frequency and change in mental health outcomes. Other studies showed that practice frequency explained changes in fatigue [36,51]. However, our results correspond to a yoga intervention study that found no significant relationship between continued yoga practice and symptoms of anxiety, depression, and fatigue [52].

### Strengths and Limitations

The study has certain limitations. The video intervention was offered to the control group after the waiting time to ensure high compliance with the study in the control group in the primary RCT [26]. It would not have been ethically justifiable to elongate the waiting period of patients in the control group because of the reduced offers during the COVID-19 pandemic and cancer patients’ reduced life expectancy. Therefore, our study on longer-term changes at the follow-up is based on an observational design without a control group. Therefore, positive changes in the fear of progression, depression symptoms, and fatigue cannot be causally attributed to the video intervention. Improvements during the study period might be caused independently by time. All outcomes were assessed with self-reported questionnaires representing a screening for mental burdens or disorders. No clinical interviews were conducted. Other confounding variables might have caused changes in mental health status, like the remission or progression of tumor disease, other current treatments, health behavior, socioeconomic status, and support, that were not assessed in our study. No adjustment for multiple comparisons was planned. However, we reported the exact *p*-values and referred results to effect sizes.

Our study included several tumor entities, but haemato-oncological tumors and breast cancer patients were highly represented. Subgroup analysis for all tumor entities was not possible because of the imbalance in the groups’ sizes. Therefore, generalizability to patients with other tumor entities is limited. Moreover, male patients and patients with lower education might be underrepresented. In line with this, other studies have reported a higher tendency for women to participate in online interventions [53]. Generally, women and breast cancer patients use alternative and complementary medicine [54]. In addition, higher educated people might be more interested and likely to use alternative and complementary medicine [54]. Thus, there could have been positive attitudes towards our intervention, which might have biased our results.

Moreover, during the recruitment, we conducted no prescreening concerning the expression of target sizes. More significant changes might have been achieved in cancer patients with higher symptom severity. However, the dropout analysis indicated that patients with more severe illnesses tended to drop out. Although dropout occurred, the proportion was within the range of other self-guided eHealth/web-based interventions [34].

Finally, information on treatment adherence (video use, exercise practice, etc.) was based on self-reporting. Therefore, retrospective bias or answers according to social desirability must be considered.

## 5. Conclusions

Symptoms of the fear of progression, depression, and fatigue decreased up to three months after the video-based intervention. However, anxiety scores remained unchanged during the study period. Focusing on post-intervention, symptoms of anxiety, depression, and fatigue were stable, while the fear of progression declined. Video usage and exercise practice during the follow-up period did not affect the outcomes. However, patients with higher needs tended to use the videos.

Our results support the further development and integration of digital interventions in psycho-oncological health care. However, further studies are required to examine and confirm the lasting effectiveness of video-based interventions. In order to improve outcomes, more interactive interventions, for example, live-guided interventions, could be established as content for further research [50]. Moreover, further studies might focus on only one or two therapeutic methods to facilitate scientific comparability and generalizability. Finally, further studies with active control conditions, extensive follow-up periods, and patients with higher symptom burdens are needed.

## Figures and Tables

**Table 1:** Assessment of treatment adherence.

Items (Number)	Response Format
Video use and practice frequency (2)	
“In the last 3 months, have you watched one or more video sequences from our study?”	“no”; “yes, single sequences”; “yes, all sequences”
“How often have you watched video sequences in the last 3 months?”	“daily”, “several times a week”, “once a week”, “seldom”, and “never”
Reasons for maintaining or quitting video usage (2)	Multiple responses
“If you continued to watch video sequences, what were your reasons?” Item stem: “Due to the videos…”:	Examples: “I feel less afraid”; “I learned to cope with my disease”; “I am less tired”; “I worry less”
“If you did not watch the video sequences any further, what were your reasons?”	Examples: “Technical struggles”; “No time to maintain video practice”; “I feel too weak”; “I did not require the videos”; “The video intervention did not help, that is the reason for quitting”

**Table 2:** Sociodemographic and clinical sample characteristics at the baseline (*n* = 155).

Characteristics	
Age in years, mean (SD); range	56.05 (12.55); 21–83
Sex, female *n* (%)	106 (68.4)
Marital status *n* (%)	
Married/living in a relationship	116 (74.9)
Single	14 (9.0)
Divorced	16 (10.3)
Widowed	6 (3.9)
Unknown	3 (1.9)
Highest graduation *n* (%)	
Less than junior (<10 years, basic secondary school)	31 (20.0)
Junior (10 years, middle-level secondary school)	61 (39.3)
Senior (high school graduate, technical college or university entrance qualification)	60 (38.7)
Other	3 (1.3)
Tumor entity *n* (%)	
Haemato-oncological tumors	49 (31.6)
Breast cancer	46 (29.7)
Gynecological tumors (other than breast cancer)	13 (8.4)
Colon carcinoma	12 (7.7)
Malignomas of skin	6 (3.9)
Pancreatic cancer	4 (2.6)
Gastric cancer	4 (2.6)
Adenocarcinoma of esophagogastric junction	4 (2.6)
Otorhinolaryngological tumors	4 (2.6)
Lung cancer	3 (1.9)
Central nerve system tumors	3 (1.9)
Gall bladder carcinomas	2 (1.3)
Other	5 (3.2)
Metastasis *n* (%)	
No	115 (74.2)
Yes	33 (21.3)
Unknown	7 (4.5)
Time since diagnosis *n* (%)	
Up to 1 month	3 (1.9)
1 to 3 months	11 (7.1)
3 to 6 months	14 (9.0)
6 to 12 months	18 (11.6)
Up to 2 years	19 (12.3)
Up to 3 years	16 (10.3)
Up to 5 years	22 (14.2)
Up to 10 years	31 (20.0)
More than 10 years	14 (9.0)
Unknown	7 (4.5)
Treatment intention, *n* (%)	
Curative	104 (67.1)
Palliative	31 (20.0)
Unknown	20 (12.9)
Therapy during study, *n* (%) ^1^	
Any therapy	84 (54.3)
Chemotherapy	46 (54.8)
Radiation therapy	11 (13.1)
Antibody therapy	31 (36.9)
Hormone therapy	14 (16.7)

^1^ Multiple therapies possible.

**Table 3:** Within-group changes in anxiety, depression, and fatigue from the baseline to the three-month follow-up.

Outcomes		Baseline/Pre-Intervention (T1)		After 3 Months (T3)		Within-Group Change				
	*n*	Mean	SD	Mean	SD	t	df	*p*-Value	SES	95%-CI
Anxiety	96	6.57	4.95	6.12	4.73	1.14	95	0.259	−0.09	−0.25 to 0.07
Fear of progression	96	31.79	9.67	29.55	9.66	3.52	95	<0.001	−0.23	−0.36 to −0.10
Depression	98	7.09	4.90	5.76	4.76	3.33	97	0.001	−0.27	−0.44 to −0.11
Fatigue	99	36.36	21.98	31.17	24.04	2.63	98	0.01	−0.24	−0.41 to −0.07

**Table 4:** Within-group changes in anxiety, depression, and fatigue from post-intervention to the three-month follow-up.

Outcomes		Post-Intervention (T2)		After 3 Months (T3)		Within-Group Change				
	*n*	Mean	SD	Mean	SD	t	df	*p*-Value	SES	95%-CI
Anxiety	98	6.21	4.87	6.00	4.73	0.74	97	0.463	−0.04	−0.16 to 0.08
Fear of progression	96	30.34	9.74	29.03	9.43	2.27	95	0.025	−0.13	−0.25 to −0.02
Depression	98	6.30	5.18	5.83	4.88	1.43	97	0.157	−0.09	−0.22 to 0.04
Fatigue	97	33.45	24.65	32.50	24.19	0.60	96	0.551	−0.04	−0.17 to 0.09

## Data Availability

The datasets analyzed during the current study are available from the corresponding author upon reasonable request.

## Supplementary Materials

The following supporting information can be downloaded at: https://www.mdpi.com/article/10.3390/ijerph20206933/s1, Table S1: Changes in anxiety, depression, and fatigue and from baseline to three-month follow-up by video-usage or exercise practice.
