# Supplementary material for: Changes of Symptoms of Anxiety, Depression, and Fatigue in Cancer Patients 3 Months after a Video-Based Intervention
Source: Int J Environ Res Public Health. 2023 Oct 17;20(20):6933. doi: 10.3390/ijerph20206933 (PMC10606592; doi:10.3390/ijerph20206933)
Supplement: Supplementary file 1 [file ijerph-20-06933-s001.zip › ijerph-2550353-supplementary.pdf]

Supplement - Table S1. Changes in anxiety, depression, and fatigue and from baseline to three-month follow-up by video-usage or exercise practice.

Outcomes	Group	n	Baseline/pre-intervention (T1)	Post-intervention (T2)	After 3 months (T3)	ANOVA Time	ANOVA Group	ANOVA Time x Group
			Mean (SD)	Mean (SD)	Mean (SD)	η^2, p	η^2, p	η^2, p
Anxiety	Users	66	6.50 (4.47)	6.30 (4.57)	6.05 (4.39)	0.008, 0.46	<0.001, 0.86	0.001, 0.85
	Non-users	29	6.45 (5.83)	5.90 (5.45)	6.00 (5.39)			
Fear of progression	Users	60	32.90 (9.14)	32.13 (10.03)	30.77 (9.77)	**0.06, 0.004**	**0.053, 0.028**	0.001, 0.89
	Non-users	31	28.87 (9.94)	27.45 (9.18)	26.42 (8.43)			
Depression	Users	59	6.80 (4.35)	6.00 (4.89)	5.49 (3.91)	**0.104, <0.001**	0.01, 0.37	0.007, 0.54
	Non-users	29	6.24 (5.22)	5.34 (4.87)	4.17 (4.03)			
Fatigue	Users	66	37.50 (19.94)	34.01 (24.86)	34.26 (23.75)	**0.049, 0.011**	0.011, 0.32	0.012, 0.32
	Non-users	28	34.82 (25.15)	30.65 (23.78)	26.09 (24.14)			
Anxiety	Practicers	38	6.95 (4.57)	6.26 (3.68)	5.92 (3.92)	0.015, 0.31	0.021, 0.20	0.008, 0.49
	Non-practicers	44	5.34 (4.69)	5.14 (5.03)	5.23 (4.62)			
Fear of progression	Practicers	34	32.35 (7.46)	30.91 (7.29)	28.85 (7.36)	**0.089, 0.001**	0.023, 0.20	0.019, 0.26
	Non-practicers	41	28.68 (9.72)	28.61 (10.69)	27.34 (9.40)			
Depression	Practicers	34	7.53 (4.15)	6.09 (4.09)	5.53 (3.45)	**0.117, <0.001**	**0.089, 0.010**	0.009, 0.52
	Non-practicers	39	4.90 (4.13)	4.10 (4.25)	3.72 (3.36)			
Fatigue	Practicers	37	40.02 (19.71)	34.23 (20.49)	33.48 (22.46)	**0.047, 0.03**	0.029, 0.13	0.005, 0.63
	Non-practicers	44	31.06 (22.02)	28.72 (25.93)	27.15 (24.52)			

ANOVA, analysis of variance; SD, standard deviation; η^2, effect size.
Bold figures indicate: significant effects ($p < 0.05$).
